# Analysis of a Customized Clutch Joint Designed for the Safety Management of an Ultrasound Robot

**DOI:** 10.3390/app9091900

**Published:** 2019-05-09

**Authors:** Shuangyi Wang, Richard James Housden, Yohan Noh, Anisha Singh, Lukas Lindenroth, Hongbin Liu, Kaspar Althoefer, Joseph Hajnal, Davinder Singh, Kawal Rhode

**Affiliations:** 1 School of Biomedical Engineering & Imaging Sciences, King’s College London, 4th Floor, Lambeth Wing, St Thomas’ Hospital, London SE1 7EH, UK; 2 Xtronics Ltd., Gravesend, Kent DA12 2AD, UK; 3 Department of Informatics, King’s College London, Strand, London WC2R 2LS, UK; 4 Faculty of Science & Engineering, Queen Mary University of London, Mile End Rd, London E1 4NS, UK

**Keywords:** ultrasound robot, robot safety, force control, ball clutch, mechanism design

## Abstract

Robotic systems have great potential to assist ultrasound (US) examination. Currently, the safety management method to limit the force that a US robot can apply mostly relies on force sensing and software-based algorithms. This causes the concern that the potential failure of sensors, electrical systems, or software could lead to patient injuries. In this paper, we investigated a customized spring-loaded ball clutch joint designed for a newly developed US robot to passively limit the force applied. The working mechanism of the clutch was modelled and the kinematic-based analysis was performed to understand the variation of the limited force at different postures of the robot. The triggering torque of the clutch was found to be 3928 N·mm, which results in the mean limited force 22.10 ± 1.76 N at the US probe end based on potential postures. The real measurement of the implemented design indicated that the limited force could be set between 17 and 24 N at the neutral posture depending on the preload. With the maximum preload, the mean limited force was found to be 21.98 ± 0.96 N based on 30 repeated measurements. The practically measured results meet the expectation from the theoretical calculation, and the resulting small variation has indicated a good repeatability of the clutch. Based on this evidence, it is concluded that the proposed clutch meets the design aim that it can limit the force applied within a safe range while at the same time ensuring that the required force is applied at different postures.

## 1 Introduction

Arm-based robotic ultrasound systems have great potential to assist extra-corporeal ultrasound (US) in small clinics and hospitals, battlefields, or remote locations. This is motivated by the deficiencies of on-site manual manipulation of hand-held probes, such as the unavailability of enough skilled sonographers for performing US examinations [1,2]. Even with skilled personnel, difficulties have also been identified in accurately holding the probe still for long periods of time using human hands when US is used for diagnosis or interventional procedure guidance, which can cause stress-related musculoskeletal complaints to the operator [3,4]. Moreover, with US-guided robotic surgery more widely performed [5], the small surgical field would potentially require another intelligent robot to hold and manipulate a US probe, rather than allowing human operators access to the surgical filed. Because of these motivations, motorizing extra-corporeal ultrasound has always been a research interest within medical robotics and ultrasound communities since the late 1990s [6]. Over the last 20 years, many systems with different mechanical configurations have been proposed, as reviewed in detail in [6,7].

The use of these robotic systems for fetal and abdominal examinations is one of the biggest research directions as they are widely used types of US scans and could include scanning of many anatomies. Examples of systems include the robotic tele-echography (TER) system proposed by Vilchis et al., which utilized a cable-driven mechanism and was clinically tested for the abdominal aortic scan [8–10]. Another well-known example is a partially motorized US robot with three degrees-of-freedom (DOFs) resulting from projects funded by the European Space Agency (ESA) [11–14]. The system was tested on both fetal and general abdominal scans. Additionally, many US robots were also tested for other applications such as in carotid artery, cardiology, and lower limb examinations. Examples of research include the works proposed by Salcudean et al. [15,16] and Abolmaesumi et al. [17–19] for image-servoing-based automated remote carotid artery examinations, the work proposed by Ma et al. for robotic-assisted multi-modality image registration developed for guiding cardiac interventional procedures [20], the work proposed by Janvier et al. for evenly spaced US scans along the length of blood vessels in lower limb as described in [21], and the work proposed by Koizumi et al. for the design and control approaches of a customized US robot to diagnose shoulder diseases [22].

In more recent years, new generations of lightweight commercially available robots have been more widely employed to explore the potentials of robotic US examination and procedure guidance. Example works include the use of an UR5 robot (Universal Robots, Odense, Denmark) from Mathiassen et al. [23], Fang et al. [24], Zhang et al. [25], and Sen et al. [26], as well as the use of a Kuka robot (Kuka Robotics, Augsburg, Germany) from Hennersperger et al. [27]. These works focus on system-level integration by making use of the matured functions of commercial robots. Application-driven advanced control methods and robotic-related image processing algorithms are the typical topics of these works. In contrast to the research carried out in the early days, the fundamental robot development itself is no longer the main focus of the research.

Among these research studies on the extra-corporeal US robot, the safe use of the robot on patients has always been an important consideration. In contrast to arm-based robots used in industry, it is impossible to keep the robot away from people as these robots are intended to directly interact with the patient’s body. For US robots, controlling and limiting the contact force that the end-effector (i.e., the US probe) can apply onto the patient’s body is crucial as inadequate forces could result in low-quality images while excessive forces could be uncomfortable for patients and cause potential injuries. Currently, the safety management method to limit the force that a US robot can apply mostly relies on force sensing and software-based algorithms, where a commercial or a customized force sensor is employed and works with various force control laws. This can be done by controlling the force based on the expert’s haptic inputs within a certain range and limits the force to a maximum allowable threshold. In the works proposed by Arbeille et al. [13,14], large-scale clinical trials of a US robot used for fetal and abdominal US examinations were introduced. The weight of the passive holder applied a force that ensured basic contact between the patient and the probe. The expert could apply additional force, which was limited to 15 N. In a subsequent study demonstrated by Essomba et al. [28], the maximum allowable force was set to 20 N. In the study proposed by Smith-Guerin et al. [29], 20 N was identified as the higher threshold as well for the cardiac, abdominal, and renal US examinations. In addition to the force control relying on expert experiences and a maximum allowable threshold, studies use metrics of US image quality as feedback to estimate the optimal force during the US acquisition, which has also become a new direction with the recent advancement of the online processing capability of US images. In a more recent study proposed by Virga et al. [30], the applied force is optimized based on the image quality and can increase up to 20 N for the abdominal aortic scan. Additionally, in the recent study proposed by Dhyani et al. [31], although the mean value of the applied force was found to be 8.2 N, the maximum force during abdominal US examination varied widely, reaching 36.5 N. There is also evidence suggesting that the required level of force is related to the body mass index (BMI) of the patient.

Although the area of sensor-based force control for US robots has been extensively discussed throughout the last decades, these control schemes have to rely on force sensing and software-based algorithms. This causes concern to both clinicians and patients that the potential failure of sensors, electrical systems, or software could lead to patient injuries, especially when US robots are practically used in remote locations where there are no engineers who can identify and deal with any technical failure. This could also be one of the reasons that only a very limited number of robotic US systems have been successfully translated clinically and commercialized. One of the most successful examples is the 3-DOF MELODY robot (MELODY, AdEchoTech, Naveil, France), resulting from ESA funded projects, which significantly relies on the on-site operator’s control of safety by manually holding the passive holder. Although US robots do not usually require a highly accurate control of force similar to the level of many surgical robots, we believe they still need some special force management features that could guarantee safety when they are used remotely without much technical support. Therefore, we believe US robots destined for clinical translation should include easily manageable mechanical safety features in addition to force sensing and software control. We consider this as a special requirement which is logically reasonable but which was missing from previous research. More generally for a commercial mechatronic system where management of force is crucial, the use of a torque-limiting joint would always be an ideal choice, although this is rarely covered in the research on US robots. This feature can be achieved by using various devices, from basic shear pins to friction and ball detent units, some with pneumatic or electrical controls. Although commercial torque-limiting devices exist in the industry, they are rarely used in the existing US robots. A possible reason is that these devices are usually expensive, un-customized, or too heavy to be included in a US robot where the aim is usually to be lightweight and inexpensive to deploy widely to remote clinics. Additionally, it is also challenging to identify certain joints kinematically where the same amount of force at the US probe end at different robot postures would result in a similar range of torque experienced at these joints.

For this paper, we investigated a customized spring-loaded ball clutch joint designed for a newly developed US robot [32] to passively limit the force applied as a more fundamental safety feature in addition to the existing sensor-based force control methods. The proposed clutch joint is inexpensive, lightweight and customized, and it can be adjusted within a certain triggering range. Based on the design, the working mechanism of the clutch was studied and modelled mathematically. More importantly, a kinematic-based analysis was performed to understand the variation of the limited force at different postures of the robot. This was to justify the proposal that with a properly designed joint configuration and kinematics, the proposed clutch could limit the force applied within a safe range while at the same time ensuring that enough required force was applied at different robot postures. The amount of triggering force at the US probe end for the implemented design was then measured at the neutral position of the robot and finally a design variation was introduced as an alternative implementation choice.

## 2 Materials and Methods

### 2.1 System Overview

**Figure 1:**
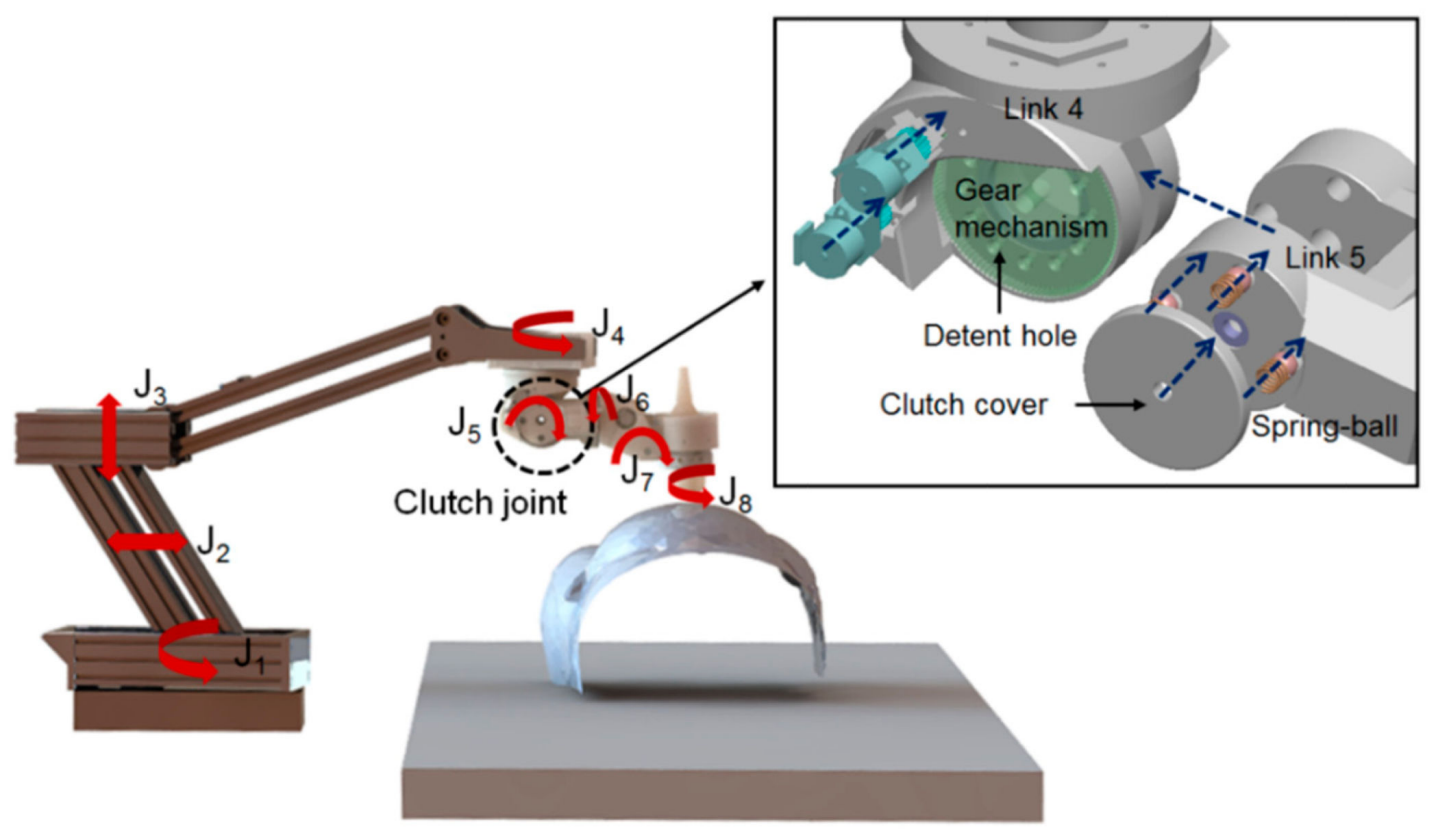
Schematic representation of the newly developed robotic ultrasound (US) system working on a fetal phantom model, with its joints labelled. The clutch design as shown in more detail in the enlarged view with this clutch mechanism and its components illustrated.

In our previous research, a robotic US system with a 5-DOF lightweight wrist unit [32] for holding and locally adjusting the probe (J_4_, J_5_, J_6_, J_7_, and J_8_), a 2-DOF two-bar arm-based set of parallel link mechanisms (J_2_, J_3_), and a 1-DOF rotational axis for global positioning (J_1_) was proposed and implemented (Figure 1). The proposed US robot was lightweight and had a smaller footprint compared to most of the commercial robotic arms used in the industry. As a result of this design, the total weight of the 5-DOF wrist was less than 2 kg including the US probe, and the length of the unit was about 25 cm. Kinematically, J_1_, J_2_, and J_3_ were used for global positioning of a US probe in a 3-DOF space. The next joints in the 5-DOF wrist, which were positioned on top of the patient’s abdomen, were used to accurately adjust the US probe’s orientation. J_4_ could rotate the next structures 360 degrees to allow the US probe to point towards different sides of the scanning area, such as the top, bottom, and sides of the abdomen. J_5_ was used to tilt the probe down to align with the surface of the scanning area. The last three orthogonal revolute joints (J_6_, J_7_, and J_8_) were used to control the tilting and axial rotation of the probe, allowing fine adjustments of the probe in a local area.

**Figure 2:**
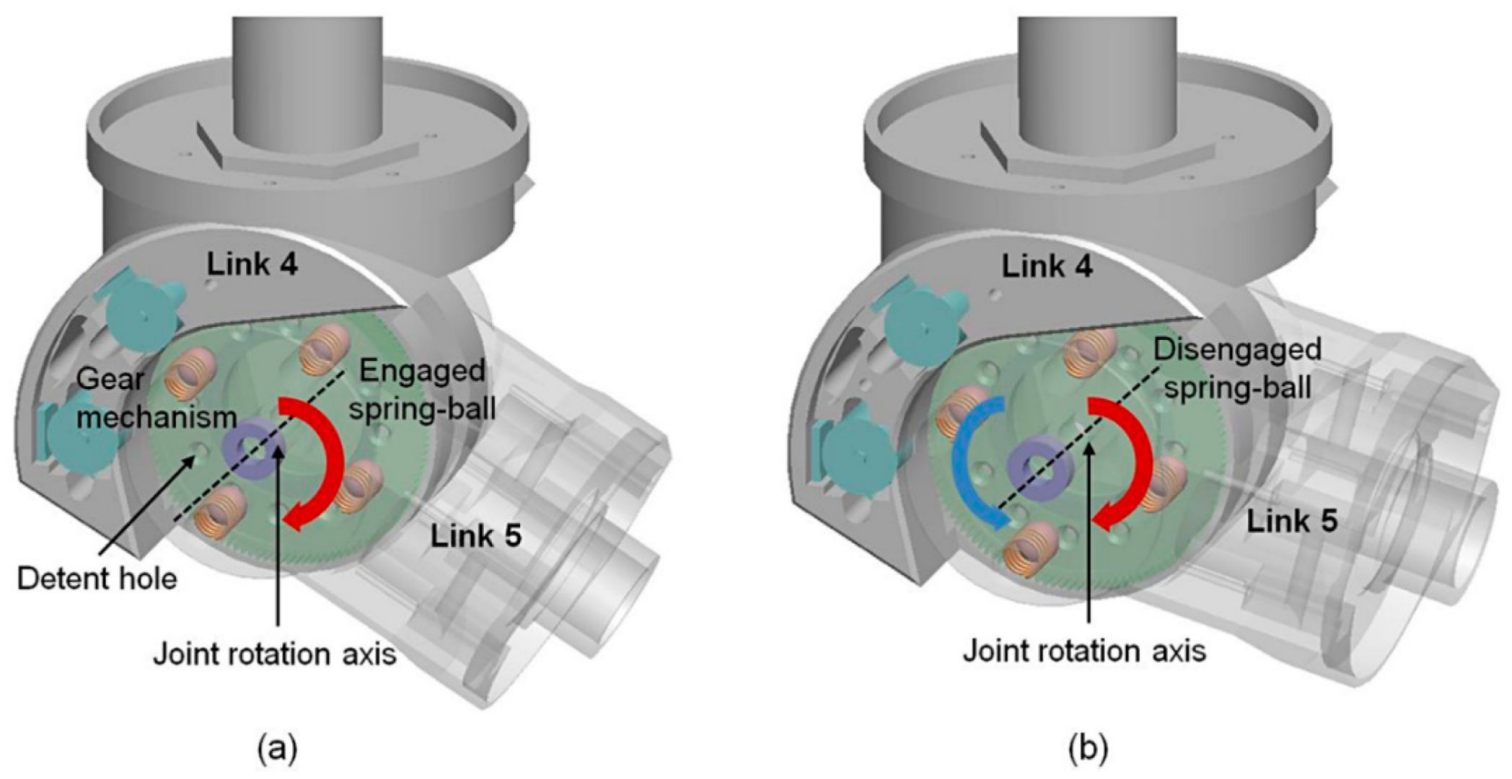
Schematic representation of the engagement and disengagement mechanisms of the clutch joint J_5_: (**a**) Links 4 and 5 are connected through the engaged spring-ball pairs and the movements of the driven gear actuate Link 5 rotating in the positive direction (red arrow); (**b**) Links 4 and 5 are disconnected with the ball moving out from the detent hole when excessive force is applied in the reverse direction (blue arrow).

In this design, the proposed clutch was implemented in J_5_ as this is the joint intended to tilt down to align the probe with the surface. This joint also had adequate space that could incorporate mechanical structures of the clutch. Moreover, the effective distance from the US probe end to the rotational center of J_5_ only varied in a limited range at different postures of the robot. Therefore, the proposed clutch could potentially limt the force applied within a safe range white at the same time ensuring that enough required force was applied at different robot postures due to the limited changes of this effective distance from the US probe end to J_5_. Detailed studies of the kinematic impacts will be presented in the following sections. For the clutch mechanism, four ball-spring pairs are inserted into the clutch detent holes in Link 5 with the two round clutch covers pushing the spring into the clutch mechanism for preloading. When the clutch is engaged, the assembled balls in Link 5, pushed by the preloaded spring, should be tightly locked inside the detent holes of the driven spur gears on Link 4 (Figure 2a). Therefore, Links 4 and 5 are connected through the engaged spring-ball pairs and the movements of the gears actuate Link 5, which forms the driving mechanism of Joint 5. However, when excessive force is exerted at the end-effector (i.e., the US probe), the clutch is triggered, which stops the torque transmission from Link 4 to Link 5, once the relative movement between the gears and Link 5 completely pushes the balls out from the detent holes (Figure 2b). If the joint continues to actuate without any intervention from the operator after the clutch is triggered, the balls re-engage and move into the next holes with the motors still running. However, the clutch is triggered again with the balls moving out from the new detent holes and the maximum force that the US probe can apply during this process is always limited to a safe threshold mechanically. Between the process of engagement and disengagement, Link 5 remains in its position with the next structures lying on top of the patient’s body. In this case, the weight of the next mechanical structures results in a pressure on the patient, but this is less than 15 N and is considered to be safe.

### 2.2 Customized Spring-Loaded Ball Clutch

**Figure 3:**
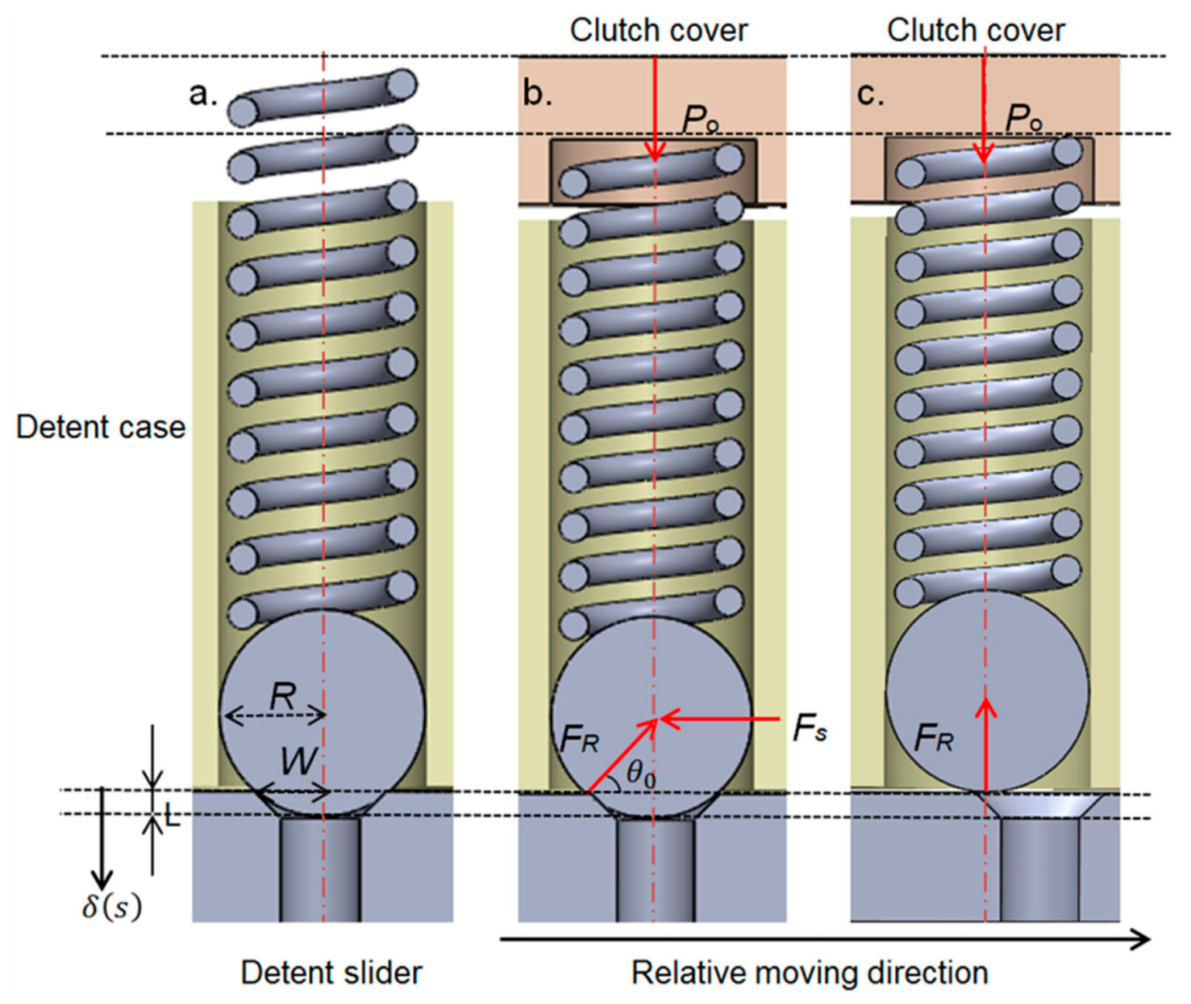
Schematic representation of an individual spring-ball detent with the geometrical parameters and the force definitions shown: (**a**) the detent assembled with the spring-ball pair, (**b**) the detent with the spring preloaded by the clutch cover, and (**c**) the state when the ball moves out of the detent hole.

To simplify the analysis and modelling of the proposed customized spring-loaded ball clutch, a small angle approximation was utilized and each spring-ball detent in the design was treated as a translational detent. This is because the amount of movement of the ball when coming out from the hole as the joint rotates in the clutch triggering stage is small, which results in the arc length subtended by the small rotational angle of J_5_ being only slightly longer than the straight line. We started analyzing the individual spring-ball detent using the exact geometry of the design (Figure 3a). When the clutch transmits torque from input to output, the detent structure is in its engaged position with the ball held by a preloaded spring compressed by the clutch cover (Figure 3b). The preload is denoted as *P*_0_ and the radius of the ball is denoted as *R*. The detent case contains the vertical compression spring with a spring constant *k*. When excessive force occurs, the detent slider slides horizontally over the detent case until the ball completely comes out from the detent hole (Figure 3c). The amount of horizontal movement in the relative movement direction during this process is denoted as *s*. These two parts develop a horizontal shear force *F_S_* between them. The inside of the slider is the conical notch with its radius denoted as *w*, which generates the reaction force *F_R_* in the angle of *θ*. The detent ball lies between the spring and the conical notch during the disengagement process. The maximum vertical displacement of the ball is as follows:

(1) $$\delta_{\max}=R-R\;\text{sin}\;\theta_{0},$$

where *θ*_0_ is the default angle in the initial position. The variable *δ*(*s*) gives the vertical displacement of the ball defined in the direction and from the starting position as shown in Figure 2a:

(2) $$\delta \left(s\right)=R-\sqrt{R^{2}-{\left(w-s\right)}^{2}}$$

As the ball comes out from the notch, the angle of the reaction force *θ* changes, which is as follows:

(3) $$\theta \left(s\right)=\arccos\left(\frac{w-s}{R}\right).$$

The vertical force *F_V_* generated by the force resulting from the compression of the spring due to the movement of the ball and the preloaded force generated from the clutch is as follows:

(4) $$F_{V}=k\left(\delta_{\text{max}}-\delta \left(s\right)\right)+P_{0}.$$

The horizontal shear force developed during the process can then be calculated as follows:

(5) $$F_{S}=\frac{F_{V}}{\tan\theta }.$$

Based on the mathematical analysis, we can calculate the side force developed during the process of triggering from the time that the ball starts moving to the time that it completely moves out of the detent hole. The maximum force during this process is treated as the triggering force of each detent hole, with the frictional force omitted in the analysis. The triggering torque of the clutch can be simply calculated based on the triggering force of each detent hole, the radial distance from the J_5_ rotational center to the detent hole, and the number of spring-ball pairs.

### 2.3 Kinematic-Based Performance Analysis

With the clutch mechanism modelled and the triggering torque of the clutch calculated, we further analyzed the performance of the clutch in terms of the resulting triggering force at the US probe end when the robot was positioned at different postures kinematically with different joint parameters, denoted as ***q***. At the triggering pose and with zero joint velocity and acceleration, the torque of the clutch joint ***Q*** comprises the torque required to hold the next links’ weight against gravity (***W_L_***(***q***)), the torque required to hold the probe’s weight against gravity (***W_P_*** (***q***)), and the joint torque due to the force applied to the US probe (***G***(***q***)):

(6) $$Q=W_{L}\left(q\right)+W_{P}\left(q\right)+G\left(q\right).$$

The torque against gravity due to the links’ weight can be estimated based on the mass center of the next links, the weight of the next links, and the angulation changes of J_5_. As the angulation changes of the last three joints only slightly influence the mass center of the next links due to the limited range of J_6_ and J_7_, the mass center was treated as a fixed point on the link and was found using CAD software (SolidWorks 2016, Dassault Systèmes, Vélizy-Villacoublay, France) with the realistic materials and weights of the components modelled. The weights of the components were separately measured using an electronic scale (Toolour, Shenzhen, Guangdong, China).

At the triggering point with different postures of the robot positioning the probe to different locations in the workspace, kinematically the torques resulted from the applied forces to the US probe and the torque required to hold the probe’s weight against gravity are different. The sum of these two terms can be calculated based on the joint configuration, and we can transfer the wrench in the probe coordinates ***g***(***q***) and the force resulting from the probe’s weight in the probe coordinates ***w***(***q***) to the torque experienced in J_5_ coordinates based on the Jacobian transpose as follows:

(7) $$W_{P}\left(q\right)+G\left(q\right)=J{\left(q\right)}^{T}(g\left(q\right)+w(q)).$$

It should be noted that the impact of the probe’s weight must be transferred to the probe coordinates based on the angulation change of J_6_ and J_7_ only. The angulation change of the axial rotation of the probe, controlled by J_8_, does not have any influence. Based on Equations (6) and (7), the triggering force experienced at the US probe end can be derived and calculated using the fixed value of ***Q*** and the changing joint parameters ***q***.

**Figure 4:**
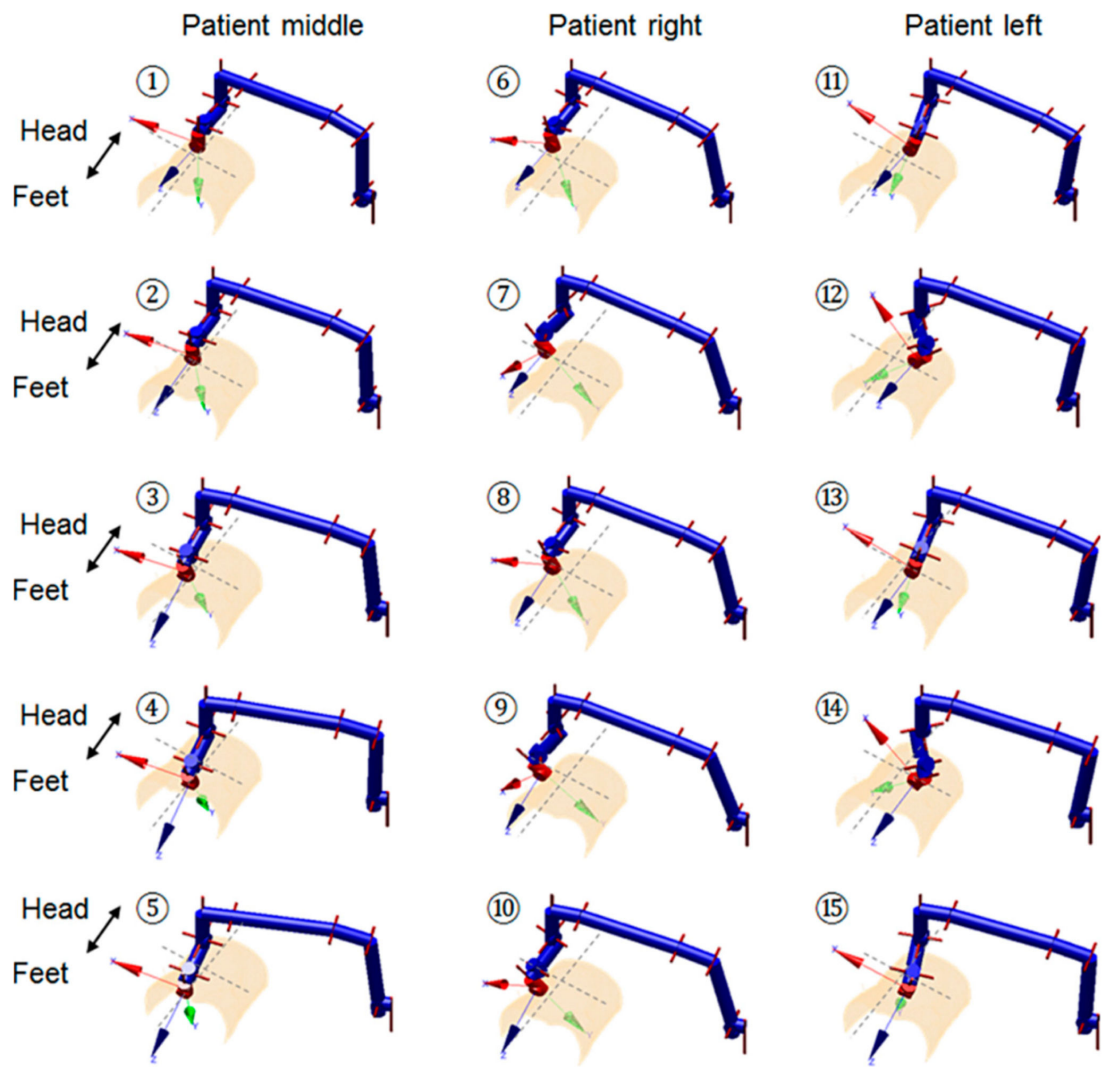
Schematic representation of the selected probe positions for the lower abdominal US scan using the robot based on the phantom geometry, with the pose numbered 1–5 for thee patient middle, 6–10 for the patient right, and 11–15 for the patient left. The joint configuration is shown for each pose.

**Figure 5:**
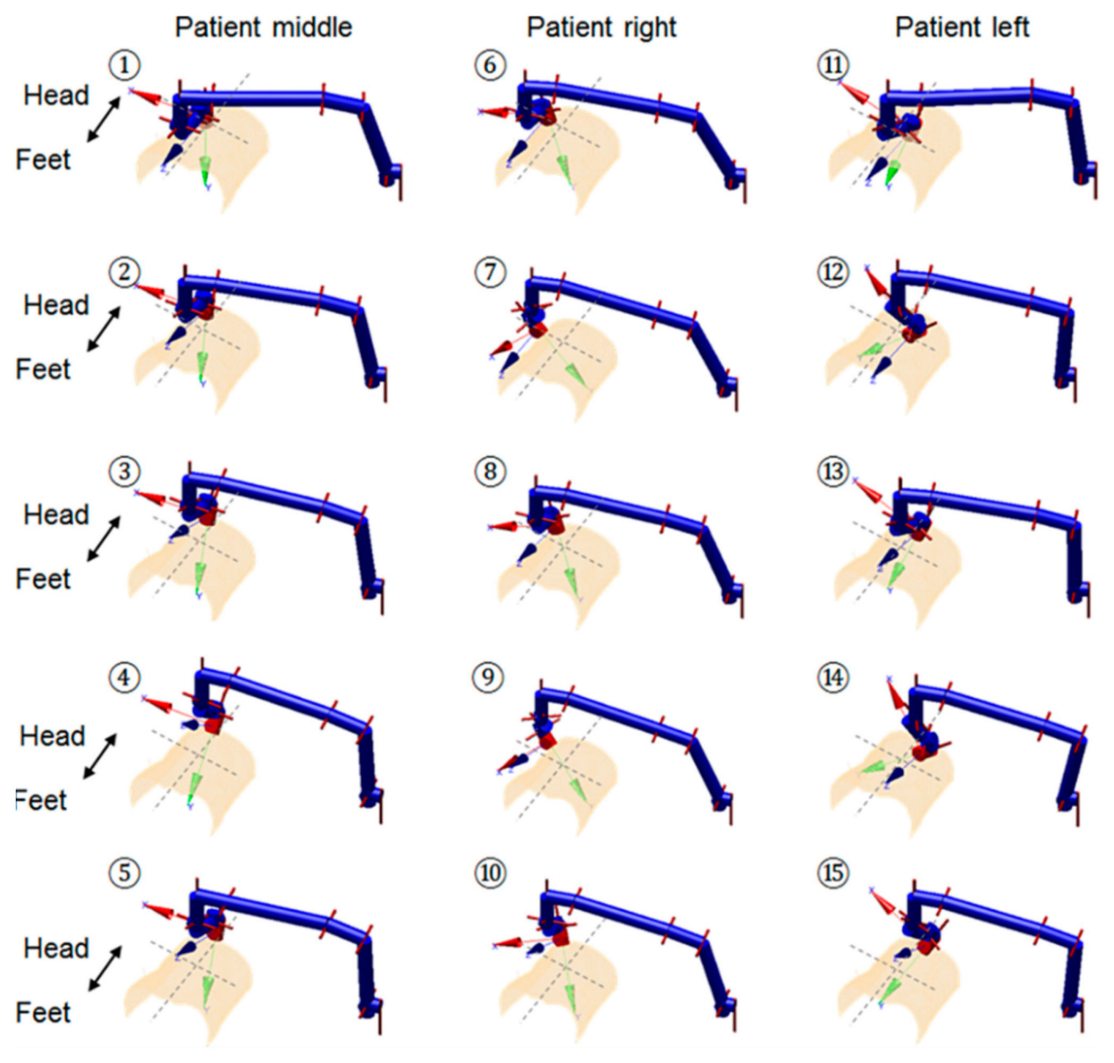
Schematic representation of the selected probe positions for the upper abdominal US scan using the robot based on the phantom geometry, with the pose numbered 1–5 for the patient middle, 6–10 for the patient right, and 11–15 for the patient left. The joint configuration is shown for each pose.

To study the impact of the joint parameters’ variation on the triggering force at the US probe end, we performed two kinematic analyses. The first analysis employed the forward kinematics of the robot and conducted an exhaustive approach covering the entire joint spaces. According to the configuration of the robot, only the angulation changes of J_5_, J_6_, and J_7_ could have impacts that influence the results calculated in Equations (6) and (7). We denote these three angles as the main tilt angle, yaw angle, and pitch angle. Different combinations of these three angles within their limits were tested and the resulting calculated forces were recorded. The second analysis used the customized closed-form inverse kinematics of the robot and positioned the US probe to clinically realistic locations that are possibly required during a real abdominal US scan using this robot. We tested this using the geometry of a fetal US phantom (SPACEFAN-ST, Kyoto Kagaku Co., Ltd., Kyoto, Japan) with 15 locations of the US probe identified for the lower and upper abdominal areas, respectively, based on the (inverse kinematics (Figures 4 and 5).

The geometry of the surface was obtained from a magnetic resonance imaging (MRI) scan of the phantom. For each of the positions defined using the inverse kinematics of the robot, the corresponding triggering force value was calculated and recorded. These 30 poses aimed to cover most of the scanning areas of the abdomen, which require different joint configurations of the robot to locate and align the US probe to be in contact with the surface.

## 3 Results and Discussion

### 3.1 Clutch Performance

Based on the analytical method for each individual spring-ball detent introduced in Section 2.2, we calculated the exact value of the triggering force of each detent and the overall triggering torque of the clutch using the following parameters from the design: *R* = 3.9 mm and *w* = 2.5 mm. The selected spring is a 10-turn music wire spring (made of high-carbon steel alloy, free length of 21.336 mm, outer diameter of 7.137 mm, inner diameter of 4.851 mm, and spring constant *k* = 9.811 N/mm). The sliding force for each spring-ball detent at the triggering point is 24.55 N, which assumes the clutch cover pushes the spring fully into the detent hole (3 mm initial compressed length of the spring) for preloading (preloaded force *P*_0_ = 29.433 N). The according triggering torque of the clutch is 3928 N.mm, with four spring-ball pairs on each side of the link.

**Figure 6:**
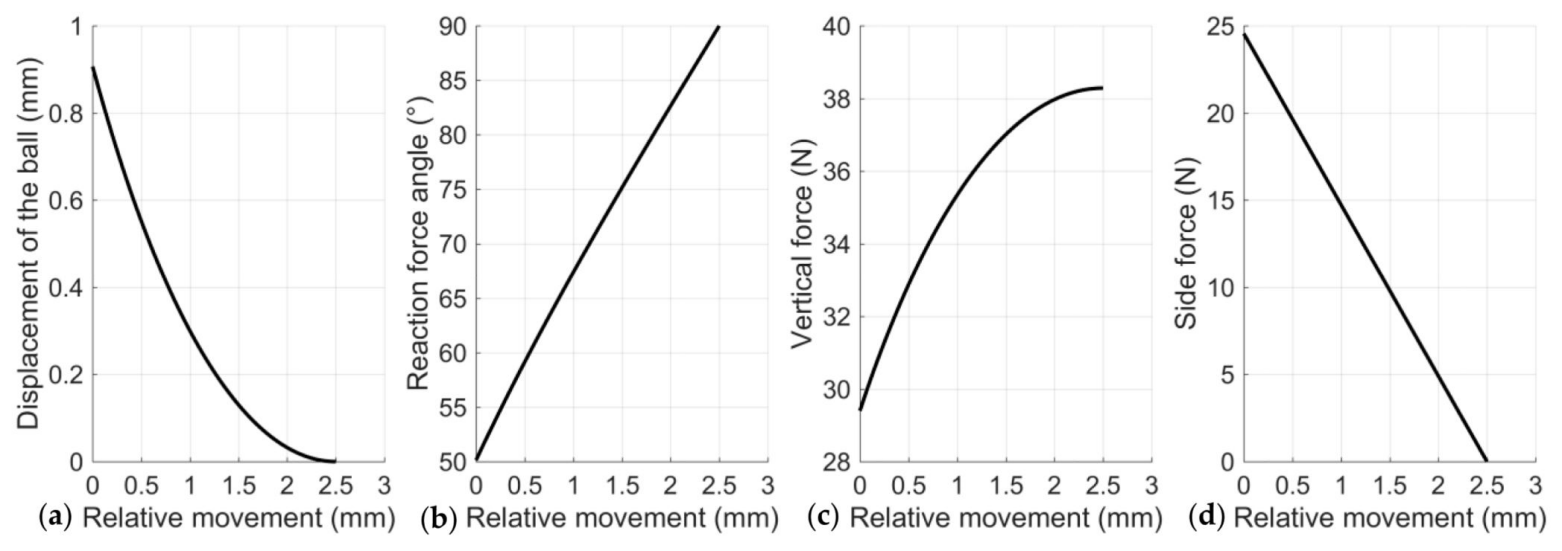
Parameter changes during the process of the ball moving out from the detent hole after the clutch is triggered based on the theoretical calculation: (**a**) the vertical displacement of the ball, (**b**) the reaction force angle, (**c**) the vertical force, and (**d**) the side force.

Analyzing the triggering process of the clutch based on Equations (1)–(5), we can also identify the change of parameters with regard to the relative horizontal movement of the ball (Figure 6), including the vertical displacement of the ball, the reaction force angle, the vertical force, and the side force. It is observed that, in our design, the side force decreases linearly from the triggering point and becomes zero force when the ball is out of the detent hole. Thus, the maximum force during the triggering process appears when the ball contacts with the notch and is about to move.

**Figure 7:**
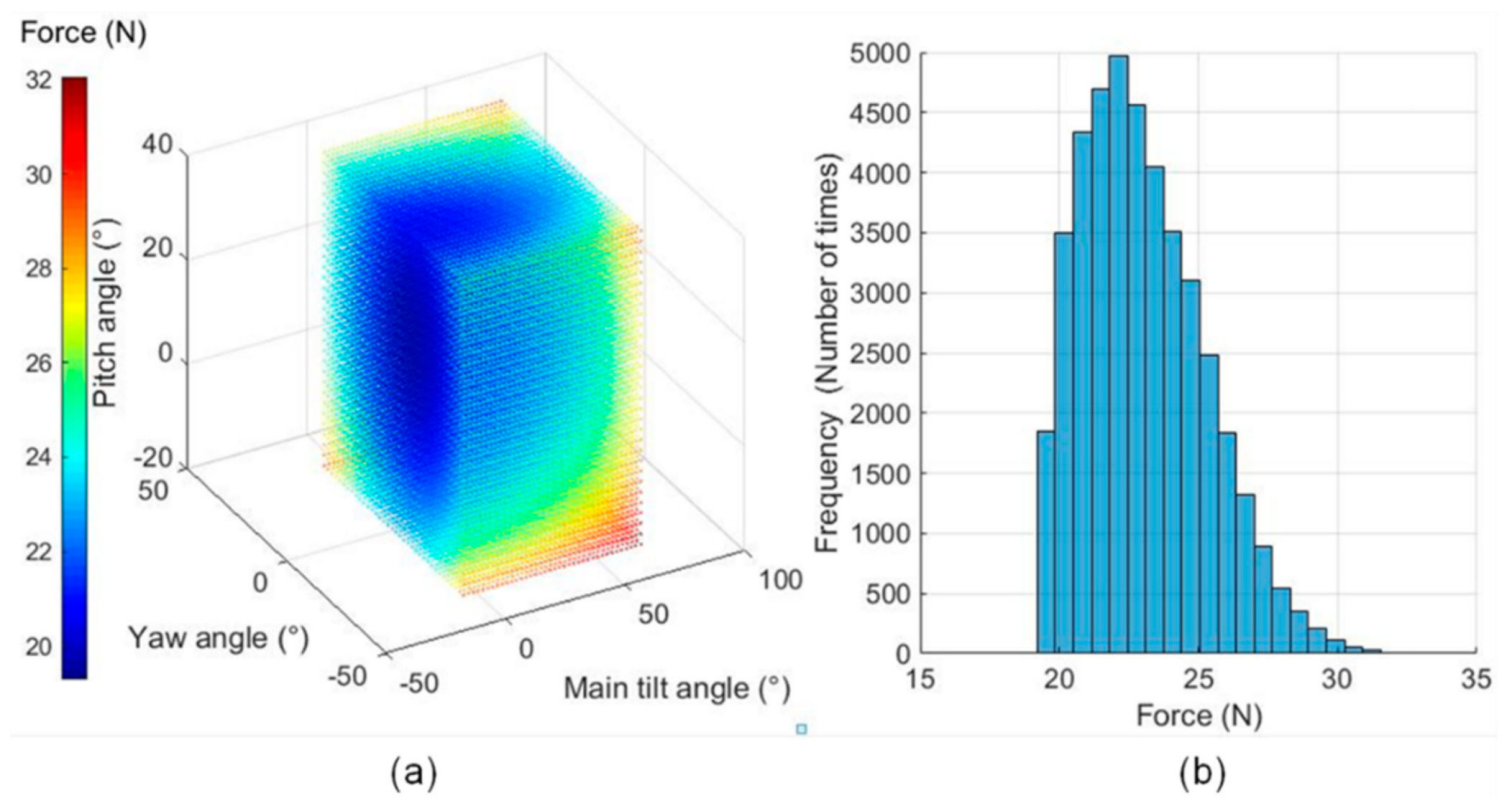
Kinematic-based clutch performance analysis using the exhaustive method: (**a**) the relationship between different joint angles (*x*, *y*, and *z* axes) and the resulting triggering force at the US probe end (color coded, in N), (**b**) the histogram of the triggering force based on the result of the exhaustive method, which covers the entire joint spaces of J_5_, J_6_, and J_7_.

For the kinematic-based analyses, the first analysis using the exhaustive method identified the relationship between the three angles (the main tilt, yaw, and pitch) and the corresponding triggering force at the US probe end (Figure 7a). This was based on the selection ranges of the main tilt angle from −5° to 70°, the yaw angle from −35° to 35°, and the pitch angle from −20° to 40° with two-degree intervals. The histogram of the triggering force is shown in Figure 7b. It is observed that the mean triggering force is 23.01 ± 2.21 N (mean ± standard deviation). The maximum triggering force is 32.06 N and the minimum triggering force is 19.29 N. Analyzing Figure 7b, it can be seen that the majority of the triggering forces are within the range of 19–26 N (91.72%) and Figure 7a shows that those high values only appear at the margin of the plot where the joint angles come to their maximum values.

Compared with the force limited values reported in the literature [13,14,28,30], we believe that the mean value of the maximum triggering force set at 23 N is reasonable in terms of safety as well as considering the adequate force for imaging. The variation of the value with the standard deviation of 2.21 N is also acceptable. This indicates that the posture changes of the robot would not significantly influence the maximum allowable force at the US probe end with a fixed design torque triggering value of the clutch joint. Although there are certain cases where the triggering force goes higher and reaches 32.06 N when the joint angles come to their maximum values, it should be noted that the joint angles rarely come to these extreme values kinematically in a real scenario. With the maximum force observed at 32.06 N, it should still be considered as safe for the patient as it is not excessive, has been observed in manual clinical scans, and would decrease to zero quickly when the ball moves out from the detent hole after the clutch is triggered.

**Figure 8:**
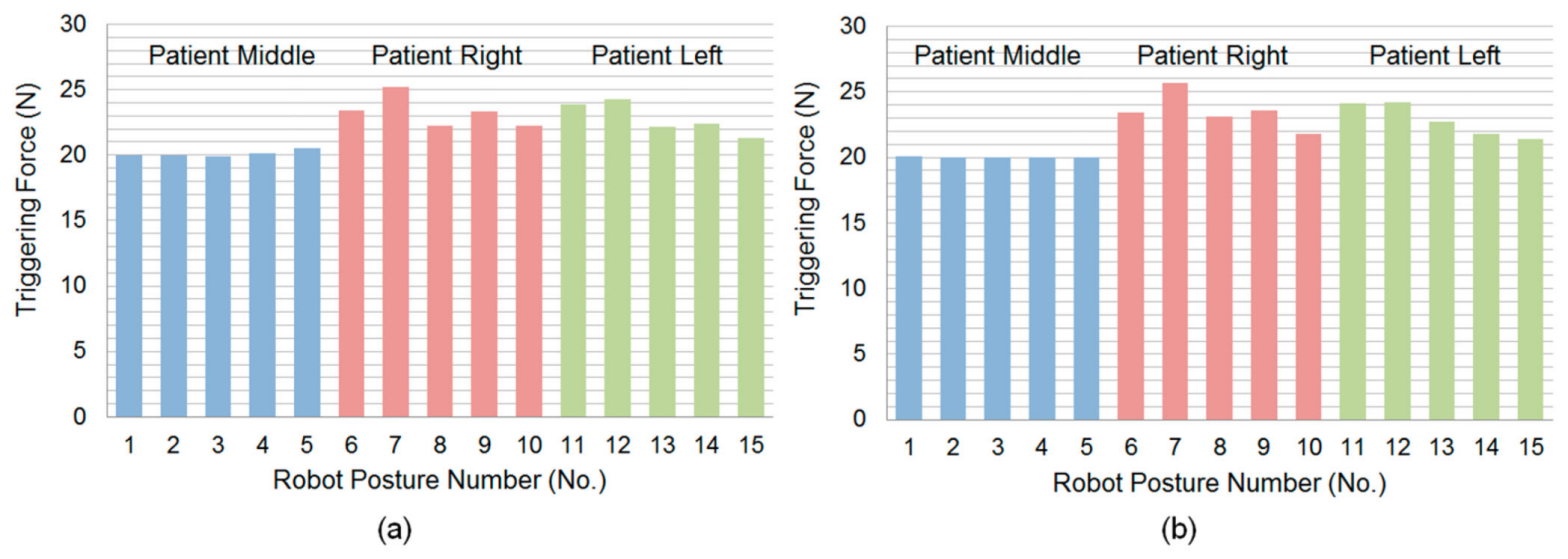
Triggering force variations at different postures of the US robot with the probe positioned at (**a**) the lower abdominal area and (**b**) the upper abdominal area, based on the joint configurations obtained from the inverse kinematics of the US robot using the geometry of a fetal phantom.

The second kinematic-based analysis of the clutch performance used more realistic joint parameters resulting from the inverse kinematics, which positions the US probe onto certain locations over an abdominal surface. The triggering force variation among those postures shown in Figures 4 and 5 are listed in Figure 8. The mean triggering force at the US probe end is found to be 22.10 ± 1.76N (mean ± standard deviation). The maximum triggering force is 25.68 N and the minimum triggering force is 19.94 N. Compared with the results from the exhaustive method, it can be seen that for the more realistic robot configurations, the mean and the minimum values of the triggering force are similar to the results from the exhaustive method, although the maximum value and the standard deviation are smallet than those found using the exhaustive method. This is because those configurations with joint angles coming to their upper limits do not usually occur realistically when the robot is scanning the abdominal area. Results from the second kinematic-based analysis further validate the successful working of the clutch mechanism concept as the identified minimum triggering value is adequate to ensure good US imaging, while the maximum triggering value is not excessive. The mean triggering value of the maximum allowable force at 22.10 N meets the expectation of the limiting force, and the standard deviation of 1.76 N indicates a relatively consistent triggering force between different postures of the robot.

### 3.2 Implementation and Practical Experiments

**Figure 9:**
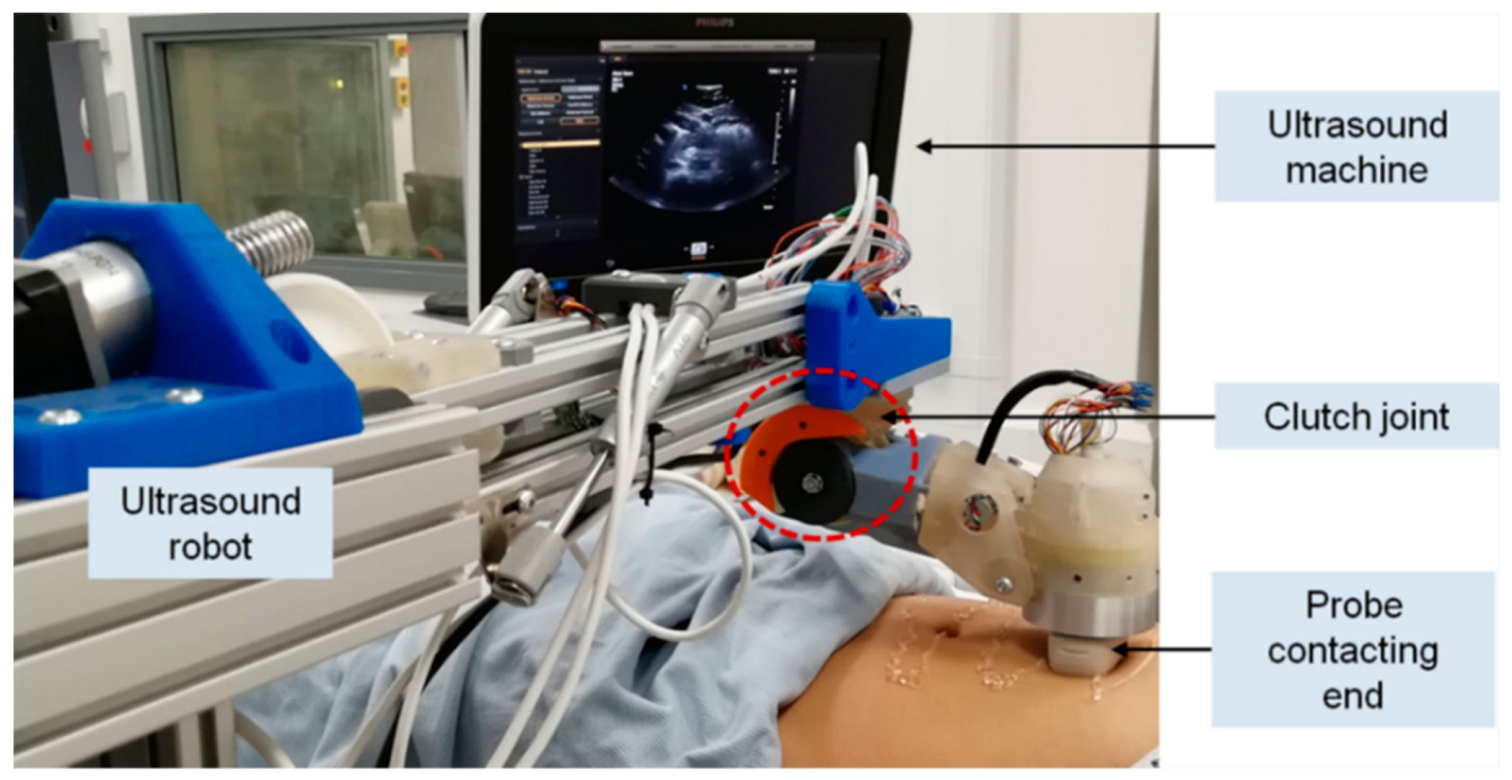
Implementation of the customized clutch based on the exact design configuration shown in the previous sections and use of the clutch joint in the pre-clinical volunteer study.

Based on the proposed design, we developed and manufactured a clutch joint which has been successfully used in the US robot and tested in a volunteer study (study title: Investigating Robotic Abdominal Ultrasound Imaging, Study reference: HR-17/18-5412, approved by the King’s College London local ethics committee, 4 April 2018). It was based on the exact design configuration shown in the previous sections. In our practical tests during the volunteer study with the setup shown in Figure 9, the clutch was triggered when a large amount of force was identified by the clinician. A manual re-engagement was then performed to reset the clutch. This verified the successful working of the clutch mechanism in a real scenario, although it only happened for a limited time and there was no report of pain from the volunteers.

**Figure 10:**
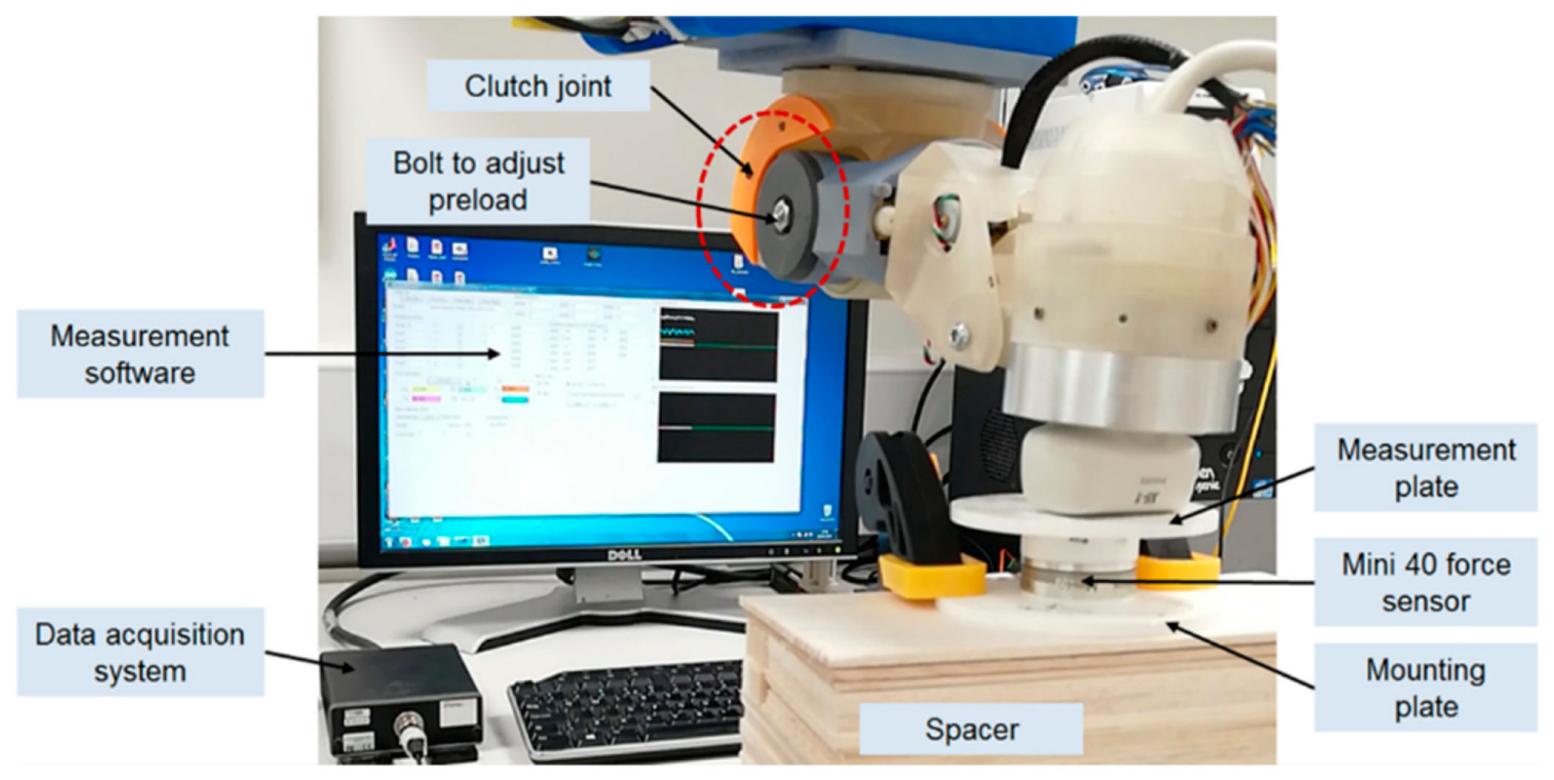
Experimental setup for the practical measurement of the triggering force using a force sensor with the robot positioned in its neutral position.

**Figure 11:**
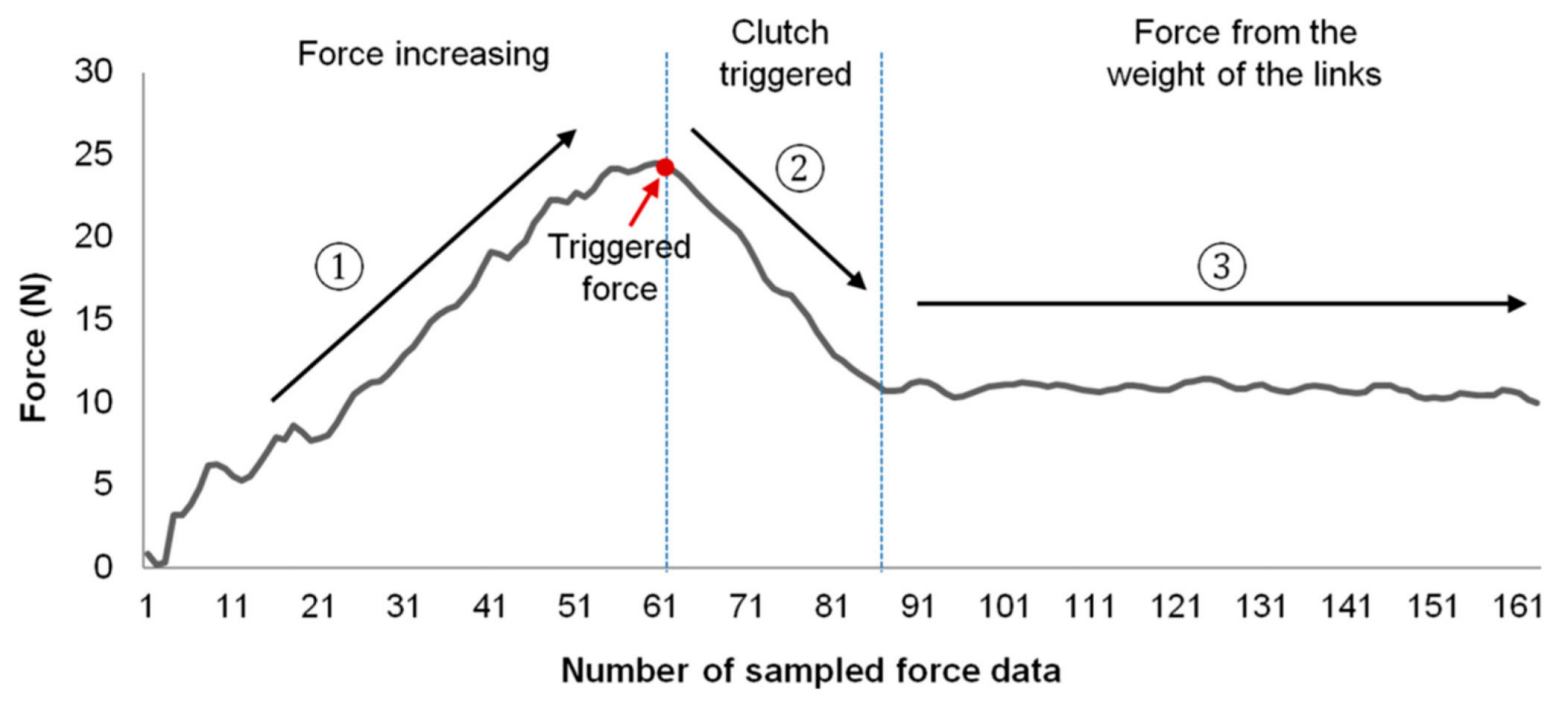
An example of the measured force data during the experiment with the force increasing at the first stage, triggering the clutch when coming to the threshold at the second stage, and eventually decreasing and settling down at a fixed value (the force resulted from the weight of the links).

To further verify the correct working of the proposed clutch and quantify the practical performance of the design, we measured the real triggering force at the US probe end when the robot was in its neutral position as the triggering force at other postures could then be estimated based on the results shown and discussed in Section 3.1. The experimental setup for the measurement is shown in Figure 10 with the robot positioned onto an experimental stage. A force sensor (Mini 40, ATI Technologies, Markham, ON, Canada) was clamped to the stage with spacers in between to locate the sensor in contact with the US probe end when the robot was in its neutral position. Two plastic plates were assembled for the mounting and measuring sides of the force sensor. During the experiment, the robot was driven downwards with increasing pressure against the force sensor. The contact vertical forces were recorded in software and the triggering values were identified when the measured force started to decrease after continuously increasing. This can be seen from an example measurement as shown in Figure 11. The triggering point can also be observed bo visually inspecting the movement of J_5_.

The measurement was repeated 30 times with the triggering force recorded for each sequence. The clutch was manually reset for each measurement after being triggered. The mean and standard deviation values of the measured triggering forces were then calculated. This was found to be 21.98 ± 0.96 N (mean ± standard deviation) when the clutch cover was pushed in to its extreme position. The assembly of the clutch cover was done using a bolt. The bolt could be slightly adjusted to fine tune the triggering force by changing the preloaded force of the spring. The adjustable range was found to be between 17 N and 24 N. The measured forces, although slightly differing from the theoretical calculated value (19.96 N at the neutral position of the robot as shown in Figure 8 for the robot configuration 1 described in Figures 4 and 5), are still very close and fall into the similar allowable range taking the preload impact into consideration. As the variation of the triggering force is limited, the similar resulting triggering force range, as identified in Figure 8, is expected for the real performance of the clutch after being assembled on the US robot. The small standard deviation of the measured triggering force indicates a relatively reliable performance of the proposed clutch with an acceptable repeatability. It is believed that these small variations within 1 N will not cause any safety concern for the reliability of the clutch.

Based on our theoretical analysis, it can be observed that the triggering force can be controlled and adjusted in the design by changing the geometry of the spring-ball detent, the size of the ball, the type of spring, and the number of balls in the clutch. However, the simplest way would be using the proposed design and changing the preload value by tightening or loosening the bolt. With more clinical evidence of the relationship between maximum allowable force and a patient’s BMI for different types of US scans, it is possible to adaptively change the clutch threshold based on the application and the specific patient. This can be achieved in the future using our current design but by increasing the preload ranges and controlling the preload more precisely with some adjustable mechanisms, for example, a linear screw mechanism where the number of turns of the screw will accurately change the preload. As the approximate correct range of the triggering force is more important than the exact value for US examinations, the proposed clutch can be easily set and regularly checked to ensure the reliable working of the system when used in practice.

It should be noted that the use of sensor-based force control methods is still strongly recommended for the applications of US robot as real-time force control can help image acquisition and ensure the good quality of images. The design and use of the clutch should be considered as a useful safety feature in addition to the conventional use of sensor-based force control as it can further guarantee safety when electronic and software failures occur. With the proposed clutch working in the engaged position, there should be no observable movements within the clutch mechanism and, therefore, Joint 5 should work reliably just as a conventional joint. If the clutch is triggered, this would not only indicate excessive force but also suggest that the existing sensor-based force control methods have failed. In this case, with the clutch disengaged, it is likely that image acquisition will be influenced as Joint 5 will not work reliably due to the slippage of the clutch. However, this scenario should already be considered as a failure case of the robot which can be observed. When this happens, the clutch should be manually reset and further inspections of the sensor-based force control should be carried out as soon as possible. From the technical analysis, the joint works normally when the clutch is engaged. Therefore, there is no direct relationship between the use of the clutch and the image acquisition with the robot, which has also been confirmed separately from our previous research with image quality analysis [33].

### 3.3 Design Variation

**Figure 12:**
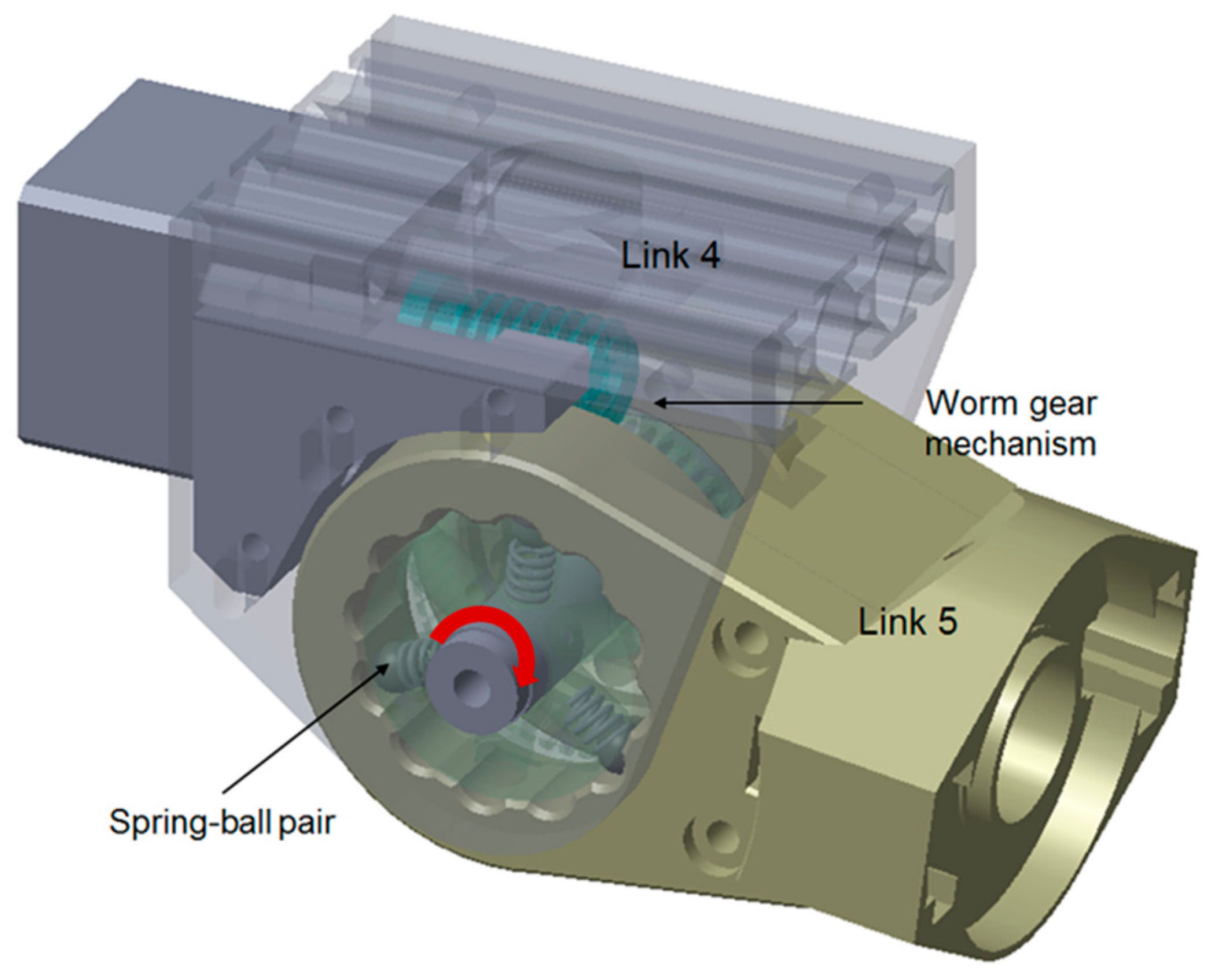
Schematic representation of the design variation based on the use of a worm gear mechanism and the axial arrangement of spring-ball detents. Three spring-ball pairs are shown for illustration, whereas more pairs can be added in practical use.

More recently, we developed a similar clutch joint with a few design changes. This was considered as a variation of the original design. Compared with the exact clutch design details presented in this paper, the variation of the original design (Figure 12) employs the same concept, but with a few changes. These include the change of the drive mechanism from spur gear to worm gear, the change of the spring-ball detent arrangement to axial, and the change of the spring type and detent angle. These changes are mainly made to adapt to the worm gear mechanism as well as improve the shaping of the link. Compared with the detailed design presented in the previous sections of this paper, this new design variation has a fixed preload value that cannot be adjusted from the aseembly of the joint. Additionally, the spring-ball pairs, the driven gear, and the next link can be pre-assembled as one modular piece. Therefore, the triggering force of the new design is less dependent on assembly and might have improved reliability.

## 4 Conclusions

This paper introduced the design, modelling, and analysis of a customized spring-loaded ball clutch joint implemented for a newly developed US robot. The aim was to propose and justify a pasive mechanical safety feature that could be used to limit the maximum force applied to the US probe end at different postures of the US robot. The calculation, analysis, and measurement results successfully justified the correct working of the designed clutch in terms of the triggering force value and its kinematic variation. Our design is customized with the clutch mechanism embedded into the bespoke link and joint, which makes it lightweight and inexpensive. The design provides a useful passive sefety feature for the US robot to limit the force applied, which is independent of the conventional force sensing system, software, and control algorithms. It is therefore concluded that the proposed clutch design is of great value and would have a very positive impact on the future development and clinical translation of US robots.
